# Total Thoracoscopic Surgery for Late Mitral Paravalvular Leakage Repair in A Beating Heart

**DOI:** 10.21470/1678-9741-2020-0692

**Published:** 2023

**Authors:** Huanan Liu, Shengjie Liao, Zhaoming Lin, Xiaoshen Zhang

**Affiliations:** 1 Department of Cardiovascular Surgery, The First Affiliated Hospital, Jinan University, Guangzhou, China.

**Keywords:** Thoracoscopy, Tricuspid Valve Insufficiency, Sternotomy, Constriction, Cardiac Surgical Procedures, Suture Techniques

## Abstract

Paravalvular leakage (PVL) after mitral valve replacement is a troublesome complication that may lead to severe symptoms and reoperation. Previous case reports on total thoracoscopic cardiac surgery without aortic cross-clamping for repairing late PVL are rare. We describe a 64-year-old man who had undergone aortic and mitral valve replacement via median sternotomy eight years earlier, and who recently developed cardiac failure due to severe tricuspid regurgitation (TR) and PVL in the posterior mitral annulus. During total thoracoscopic surgery with using the beating heart technique, direct closure of the PVL was achieved via pledgeted mattress sutures, and tricuspid valvuloplasty was routinely performed to treat TR. This case indicated that total thoracoscopic surgery on a beating heart may be an excellent option for treating PVL concomitant with TR.

## INTRODUCTION

Paravalvular leakage (PVL) is a well-known complication following valve replacement with an incidence between 5% and 32%^[1,2]^. For patients who develop PVL after mitral valve replacement, aggressive surgical interventions are recommended because the long-term clinical outcomes of other treatments may be less favorable, such as lower event-free survival rates^[2]^. Nevertheless, the failure rate of valve re-replacement is higher than that of direct suture repair of the leakage site^[3]^. Median resternotomy for redo valve surgery often entails a high risk of hemorrhage and structural damage. The beating-heart technique without aortic cross-clamping and cardiac arrest may reduce the possibility of myocardial ischemia-reperfusion injury^[4]^. Previous case reports on totally thoracoscopic surgery without aortic cross-clamping for repair of late PVL are rare. In this paper, we aimed to show the technical approach for total thoracoscopic surgical repair of the mitral valve with the beating-heart technique.

## CASE PRESENTATION

An ethical approval was obtained from the institutional board and authorities. A 64-year-old man had a history of biological valve replacement for aortic and mitral regurgitation via a median sternotomy eight years earlier at another hospital. His postoperative course was uneventful until 2017, when he developed exertional dyspnea. With his condition deteriorating, he was transferred to our hospital.

**Fig. 1:**
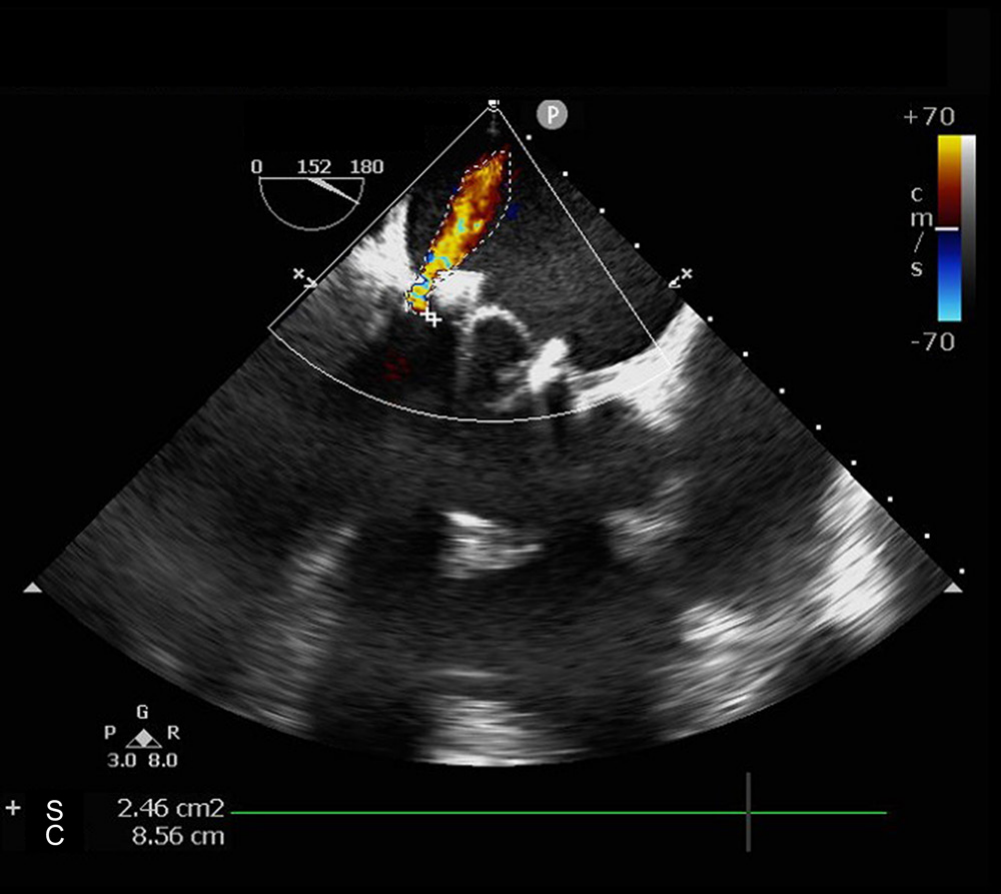
Preoperative transesophageal echocardiography of this patient showing paravalvular leakage in the mitral biological valve.

**Fig. 2:**
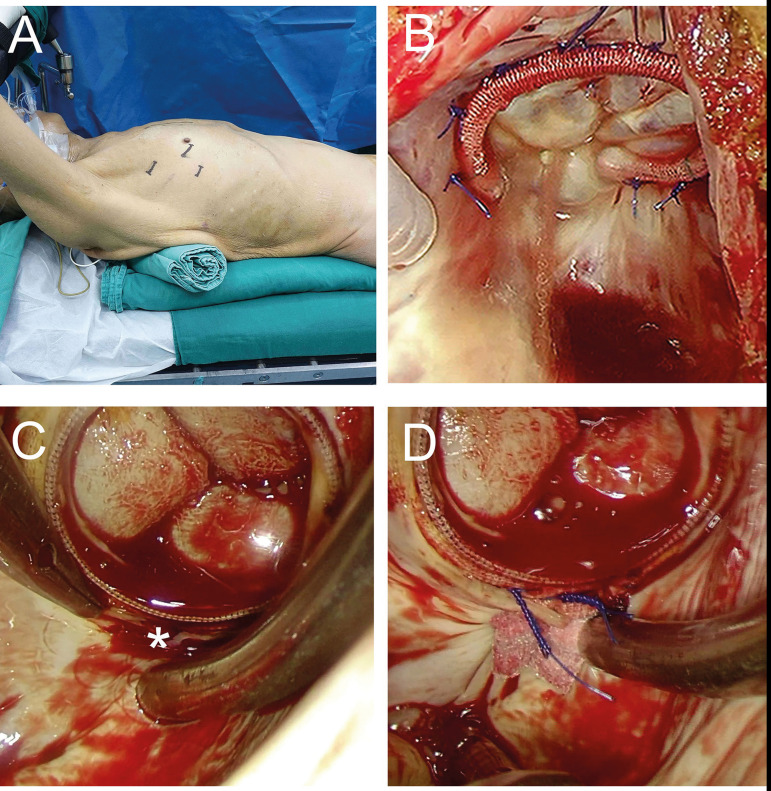
Views during thoracoscopy-assisted and beating-heart surgery. (A) Positions of skin incisions. (B) Minimally invasive tricuspid valvuloplasty. (C) Leakage site in the mitral annulus (asterisk); (D) Direct closure of mitral paravalvular leakage using pledgeted mattress sutures.

The patient developed severe heart failure (NYHA class IV). On physical examination, a diastolic rumbling murmur (Levine II/III) was heard over the left sternal border. Chest radiography revealed cardiomegaly with a cardiothoracic ratio of 54%. Preoperative transthoracic echocardiography (TTE) showed that the left ventricular ejection fraction (LVEF) was 31%, and severe tricuspid regurgitation (TR) was detected. The left atrium (49 mm) was enlarged. The left ventricular end-diastolic and end-systolic diameters were 48 and 34 mm, respectively, with mild PVL at the mitral position. The estimated pulmonary artery pressure, measured by TTE, was normal. Transesophageal echocardiography (TEE) confirmed PVL in the posterior mitral annulus (Figure 1). Coronary angiography and chest computed tomography were unremarkable. We performed total thoracoscopic surgery to treat PVL and TR with the beating-heart technique (Figure 2 and Video).

**Video:**
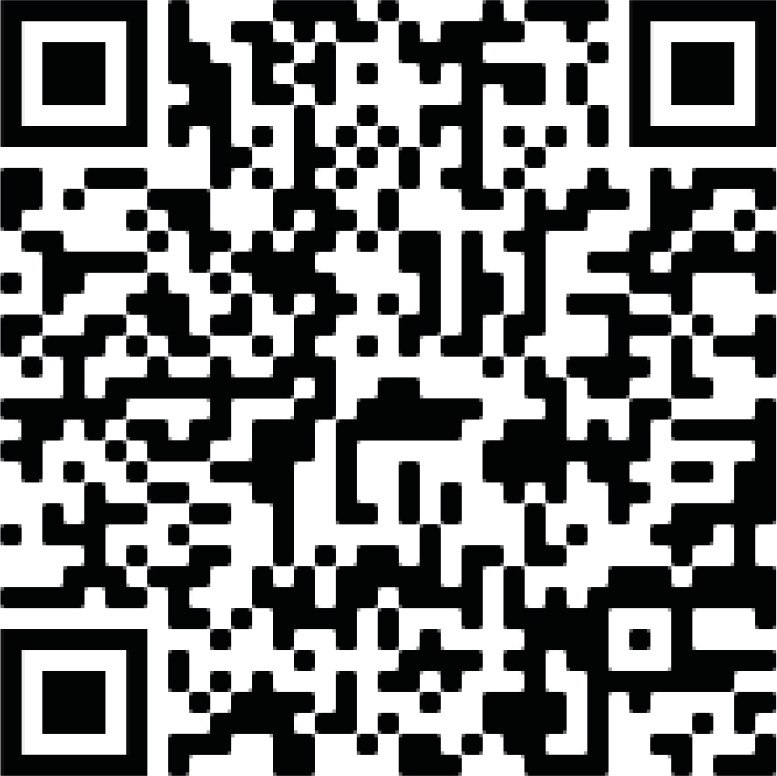
Surgical procedure for tricuspid regurgitation and paravalvular leakage at the posterior mitral annulus.

## TECHNICAL DESCRIPTION

The patient was intubated via a double-lumen endotracheal tube and placed under combined intravenous-inhalation anesthesia. He was then placed in the supine position with the right chest slightly elevated. Cardiopulmonary bypass (CPB) was established via right femoral arterial and venous cannulation. Three incisions were made for the insertion of working ports (Figure 2A). A camera port, which functioned as the entrance for the thoracoscope (Olympus, Japan), was inserted along the right midaxillary line at the 5^th^ intercostal space. An assist port was inserted on the right midaxillary line at the 3^rd^ intercostal space. This port was used for placement of the superior vena cava drainage tube and left atrial suction tube during the operation. The 3^rd^ port was inserted on the right anterior axillary line at the 4^th^ intercostal space for placement of surgical instruments, such as scissors and needle holders.

**Fig. 3:**
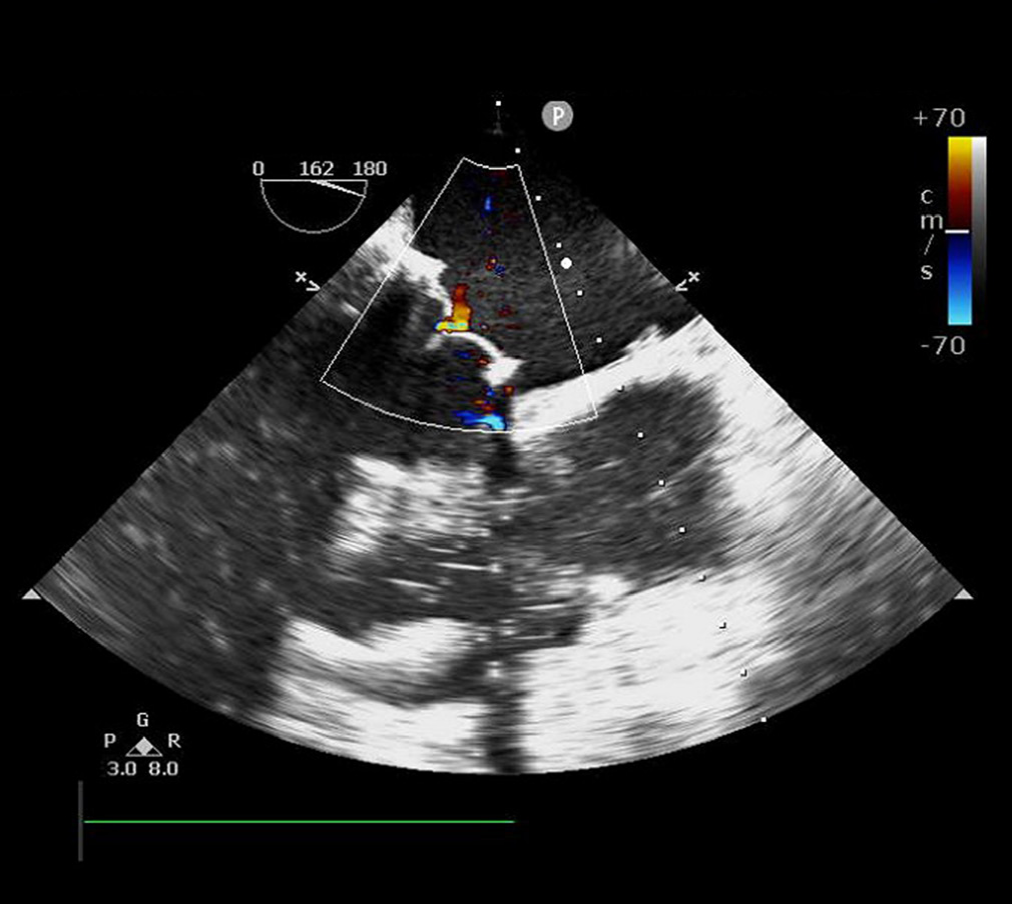
Intraoperative transesophageal echocardiography showing no leakage.

After a careful dissection of a stubborn pericardial adhesion, a Contour 3D annuloplasty ring (Medtronic, CA, USA) was accordingly implanted in the tricuspid annulus via a right atriotomy (Figure 2B). Then, a drainage tube was inserted into the superior vena cava, and an incision was made in the atrial septum. The patient was placed in the head-down position, and carbon dioxide was continuously insufflated into the chest throughout the redo surgery to displace intracardiac air. After a left venting tube (WEGO, Shandong, China) was inserted into the pulmonary vein, the anterior wall of the left atrium was lifted by a blade retractor, which penetrated parasternally through the 4^th^ intercostal space. The prosthetic mitral valve was well exposed and located without any disturbance of movement. However, a crack was found in the posterior mitral annulus (6-7 o’clock according to the surgeon’s view), which had led to the PVL (Figure 2C). Two pairs of stitches were placed through a fold of the polyester cardiovascular patch (Chest, Shanghai, China) and were then brought directly into the sewing ring. When the tying was completed, direct closure of the PVL was achieved by using pledgeted mattress sutures (Figure 2D). The incisions in the atrial septum and the right atrium were then sutured with 4-0 Prolene (Ethicon). At the same time, the air was drawn out and exhausted to avoid air embolisms. After the patient was weaned from CPB, intraoperative TEE demonstrated the restoration of mitral and tricuspid valve competences (Figure 3). A chest drainage tube was placed through the camera port. Follow-up TTE revealed an LVEF of 54%. The postoperative recovery was uneventful and the patient was discharged 12 days after surgery.

## COMMENT

PVL will complicate the postoperative course and can manifest as hemolytic anemia and/or heart failure. Mitral valves are more susceptible to symptomatic paravalvular leaks than other valves, and prosthetic leaks occur mostly in the early postoperative period^[5]^. Although paravalvular leaks are more commonly seen in patients with mechanical valves than in those with bioprostheses, PVL in the biological valve was found in the patient in our study eight years after mitral valve replacement. Various causes can contribute to the development of PVL, such as infection, annular calcification and technical aspects of the replacement surgery. In our case, the patient showed no signs of infection or other systemic disorders that could have resulted in tissue fragility after we examined the laboratory data preoperatively and scrutinized the mitral annulus intraoperatively. Despite the lack of a pathological examination of the native tissue, the stitches and annulus showed no disruption, and the tissue was minimally calcified. The cause of late PVL in this case was speculated to be the liability of the sewing cuff to become detached from the native tissue at the weakest tying site of the initial surgery due to the accumulated stress in the mitral annulus.

Medical treatment for clinically significant PVLs is mainly palliative. However, the gold standard treatment for severely symptomatic PVLs is surgical closure, including re-replacement and local repair. Because suture repair might prevent the development of new leaks better than a repetition of the valve replacement surgery and the previous sutures remained attached to the sewing cuff, we considered local repair of the PVL through the atrial septum to be the best option. Previously, full-thickness atrial septal tissue adjacent to the sewing cuff had been used for repairing anterior mitral PVLs, and satisfactory durability was achieved^[3]^. However, this technique was adopted only in the anterior side of the mitral valve. Pledgeted mattress sutures in the preserved cuff were reported to be effective for repairing PVLs under median resternotomy and cardiac arrest^[6]^. In this case, we managed to repair the mitral PVL by placing two pairs of mattress sutures in a beating heart under total thoracoscopic surgery. No residual leakage was observed. It is worth noting that this repair technique could be more suitable in non-infective mild and moderate PVLs^[7]^. Fresh autologous pericardium is highly recommended as a patch material, but stubborn pericardial adhesion makes it inapplicable during redo cardiac surgery.

Percutaneous repair of PVL has been reported as an alternative approach to surgery for patients who are not suitable for reoperation^[8]^. However, this procedure requires a transcatheter device specifically designed to achieve promising results. In addition, the patient in our study showed severe tricuspid regurgitation, which also demanded valvuloplasty. Thus, reoperation was quite necessary.

The reoperation was primarily performed via median sternotomy. Postoperative adhesions between the sternum and cardiac structures increased the risk of hemorrhage during resternotomy and subsequent dissection. Video-assisted thoracoscopic cardiac surgery is believed to have advantages over conventional open-heart surgery, including minimal access, less postoperative pain, a decreased risk of infection and quicker recovery, which contribute to lower operative mortality and greater patient satisfaction^[9]^. Here, we applied total thoracoscopic surgery techniques for treating PVL and TR. This approach yielded excellent exposure of the mitral prosthesis and the tricuspid valve.

Moreover, we used the beating-heart technique instead of cardioplegic arrest to perform valve surgery. The avoidance of aortic cross-clamping and the maintenance of continuous coronary perfusion lowered the risks of myocardial ischemia and reperfusion injury. Because we used the beating-heart technique, we observed the absence of periprosthetic leakage once the pledgeted mattress sutures were placed. Regarding air embolisms, we adopted deairing techniques with the patient in the head-down position, injection of carbon dioxide and left atrial venting. No neurological complications caused by air embolisms were observed using the aforementioned methods, confirming the results from other studies^[4]^.

## CONCLUSION

The outcome of this patient, who underwent totally thoracoscopic repair of a late PVL on a perfused beating heart, was favorable. This procedure is an excellent option for treating PVL concomitant with TR and we believe that some patients can benefit from this technique.
